# What evidence exists on the performance of nature-based solutions interventions for coastal protection in biogenic, shallow ecosystems? A systematic map protocol

**DOI:** 10.1186/s13750-023-00303-4

**Published:** 2023-05-22

**Authors:** Avery B. Paxton, Trevor N. Riley, Camille L. Steenrod, Carter S. Smith, Y. Stacy Zhang, Rachel K. Gittman, Brian R. Silliman, Christine A. Buckel, T. Shay Viehman, Brandon J. Puckett, Jenny Davis

**Affiliations:** 1 National Centers for Coastal Ocean Science, National Ocean Service, National Oceanic and Atmospheric Administration, 101 Pivers Island Road, Beaufort, NC 28516 USA; 2 Central Library, Office of Science Support, Oceanic and Atmospheric Research, National Oceanic and Atmospheric Administration, 1315 East-West Highway, Silver Spring, MD 20910 USA; 3 CSS Inc., 10301 Democracy Lane, Suite 300, Fairfax, VA 22030 USA; 4 Duke University Marine Lab, 135 Marine Lab Road, Beaufort, NC 28516 USA; 5 Department of Marine, Earth, and Atmospheric Sciences, North Carolina State University, 2800 Faucette Drive, Raleigh, NC 27695 USA; 6 Department of Biology, East Carolina University, 101 E. 10th Street, Greenville, NC 27858 USA; 7 Coastal Studies Institute, East Carolina University, 850 NC 345, Wanchese, NC 27981 USA

**Keywords:** Coastal hazards, Coastal resilience, Ecological engineering, Ecosystem-based adaptation, Green infrastructure, Living shoreline, Natural and nature-based feature, Natural infrastructure, Nature-based infrastructure

## Abstract

**Background:**

Anthropogenic pressures and climate change threaten the capacity of ecosystems to deliver a variety of services, including protecting coastal communities from hazards like flooding and erosion. Human interventions aim to buffer against or overcome these threats by providing physical protection for existing coastal infrastructure and communities, along with added ecological, social, or economic co-benefits. These interventions are a type of nature-based solution (NBS), broadly defined as actions working with nature to address societal challenges while also providing benefits for human well-being, biodiversity, and resilience. Despite the increasing popularity of NBS for coastal protection, sometimes in lieu of traditional hardened shorelines (e.g., oyster reefs instead of bulkheads), gaps remain in our understanding of whether common NBS interventions for coastal protection perform as intended. To help fill these knowledge gaps, we aim to identify, collate, and map the evidence base surrounding the performance of active NBS interventions related to coastal protection across a suite of ecological, physical, social, and economic outcomes in salt marsh, seagrass, kelp, mangrove, shellfish reef, and coral reef systems. The resulting evidence base will highlight the current knowledge on NBS performance and inform future uses of NBS meant for coastal protection.

**Methods:**

Searches for primary literature on performance of NBS for coastal protection in shallow, biogenic ecosystems will be conducted using a predefined list of indexing platforms, bibliographic databases, open discovery citation indexes, and organizational databases and websites, as well as an online search engine and novel literature discovery tool. All searches will be conducted in English and will be restricted to literature published from 1980 to present. Resulting literature will be screened against set inclusion criteria (i.e., population, intervention, outcome, study type) at the level of title and abstract followed by full text. Screening will be facilitated by a web-based active learning tool that incorporates user feedback via machine learning to prioritize articles for review. Metadata will be extracted from articles that meet inclusion criteria and summarized in a narrative report detailing the distribution and abundance of evidence surrounding NBS performance, including evidence clusters, evidence gaps, and the precision and sensitivity of the search strategy.

**Supplementary Information:**

The online version contains supplementary material available at 10.1186/s13750-023-00303-4.

## Background

Healthy coastal ecosystems provide services, ranging from food provisioning and carbon sequestration to nutrient cycling and water purification [2, 54]. These ecosystems, including salt marshes, seagrasses, mangroves, kelp forests, shellfish reefs, and coral reefs, also serve to buffer communities from coastal hazards by reducing physical impacts, such as shoreline erosion, wave energy [2, 80], and storm surge [38]. For example, wave height can be reduced by salt marsh vegetation by 60% [58], fringing oyster reefs by 30–50% [92], and coral reefs by 84% [27]. The ability of coastal systems to dampen wave energy can reduce erosion [11, 71] and in some cases, trigger a shift from coastal erosion or shoreline retreat to accretion [55]. Attenuation of storm surge by mangrove forests [94] and marshes [1, 30] may also contribute to coastal protection by substantially decreasing the vulnerability of coastal communities.

Combined impacts from anthropogenic pressures and climate change threaten the capacity of coastal ecosystems to protect communities from hazards. Anthropogenic threats, including overexploitation, pollution, development, and habitat degradation, have triggered losses in habitat coverage across many coastal ecosystems, with global declines measuring 85% in oyster reefs [3], ~ 19–29% in seagrass meadows [18, 88], ~ 50% in coral reefs [19], 42% in salt marshes [31], 35% or higher in mangroves [34, 65, 87], and also prevalent in kelp [20, 50]. Losses in habitat cover directly remove the structural components of the ecosystem (e.g., vegetation, reef substrate) that are largely responsible for coastal protection. Experimental evidence suggests that removing marsh vegetation limits the ability of marshes to reduce wave energy [58], and modeling efforts demonstrate linkages between coral reef loss and increases in wave energy [74]. As habitats are degraded or lost, their ability to provide ecosystem services, such as flood protection, is expected to decline [21, 79]. Mangrove deforestation in Myanmar, for example, decreased the total value of mangrove-associated ecosystem services by almost 30% over 14 years, of which almost 11% was attributed to a loss of coastal protection services [21].

With effects from climate change, including rising sea levels, changing precipitation patterns, intensifying storms, and increasing temperatures, the capacity of natural coastal ecosystems to protect communities can be overwhelmed or reduced, especially in systems experiencing effects of heightened anthropogenic activity [80]. Projections under these extreme scenarios suggest that previously degraded coastal ecosystems will experience further changes, loss, and degradation [17, 29, 75, 93]. For example, mangroves may experience higher rates of erosion as wave heights increase with climate change [75], while coral reef regeneration may be impaired after storms when combined with stressors from anthropogenic activities [29]. When extreme events overcome the natural protection afforded by ecosystems, it can impose direct threats to and increase the vulnerability of coastal communities [59]. For instance, storm surge, which has already been responsible for almost half of the fatalities from tropical cyclones in the United States from 1963 to 2012 [66], is expected to cause more fatalities as humans continue to migrate to coastal areas and the percentage of urban land at low elevations along the coast increases [39]. Additionally, populations within coastal communities that are unwilling or unable to move may incur greater risks as flooding increases [57].

To improve coastal protection, resource managers, governments, local municipalities, tribal nations, military installations, non-governmental organizations, and private property owners are increasingly turning to nature-based solutions. Nature-based solutions (NBS) are broadly defined as “actions to protect, conserve, restore, and sustainably use and manage natural or modified terrestrial, freshwater, coastal, and marine ecosystems to address social, economic, and environmental challenges effectively and adaptively, while simultaneously providing human well-being, ecosystem services, resilience, and biodiversity benefits” [85]. Phrased more concisely, NBS are “actions that involve people working with nature, as part of nature, to address societal challenges, providing benefits for both human well-being and biodiversity” [72]. NBS is an umbrella term [60] that includes measures like green infrastructure, natural and nature-based features [4], nature-based infrastructure [78], natural infrastructure [25], nature-climate solutions [37], and ecosystem-based adaptation [13]. *Here, we focus on the subset of active NBS interventions used to improve coastal resilience to hazards by providing physical protective services, such as wave attenuation, flood reduction, and sediment stabilization.*

Active nature-based solutions for coastal protection can come in a variety of forms and may include the creation or restoration of a variety of ecosystems with or without the inclusion of engineered structural components. What these NBS techniques all have in common is the goal of providing some kind of physical protective service, such as reduced erosion and inundation, while also providing ecological co-benefits. Ecological co-benefits include, but are not limited to: increased biodiversity, improved water quality, and habitat enhancement, as well as the ability to adapt to and keep pace with stressors like sea level rise, that “gray” infrastructure (e.g., seawalls, bulkheads) either do not provide or exacerbate (e.g., block connectivity) [4, 5, 78]. Additional social benefits of NBS projects may include increased tourism [53], improvements in the aesthetic value of coastal habitats, and expanded access to cultural activities through environmental programs [14]. Economically, NBS often provide more cost-effective solutions for inundation protection, as they can eliminate typical maintenance costs and responsibilities associated with “gray” infrastructure [25, 76, 83], effectively preventing billions of dollars in flood-associated losses and repairs [67]. Although the economic and social benefits of NBS are often less thoroughly assessed than ecological benefits [77], primarily due to limited socio-economic data availability and difficulties in data collection [63], understanding the suite of benefits NBS provide can help recognize the full potential of NBS projects for coastal protection [83].

Growing evidence that NBS can provide coastal protection (physical benefits) and other valuable ecological, economic, and social co-benefits if strategically designed, placed, constructed, and managed has spurred international efforts to broadly adopt NBS for protecting coastal communities and investments from threats of climate change and associated hazards [46, 48, 49, 84]. The United Nations and International Union for Conservation of Nature (IUCN), heralding the 2020s as the “Decade on Ecosystem Restoration,” called for approaches to reduce ecosystem degradation, one of which was nature-based solutions [86]. In the United States (US), this call has been met with landmark federal funding initiatives to boost the widespread use of NBS. Most recently, the US Infrastructure Investment and Jobs Act (IIJA, November 2021) allocated $47 billion for climate resilience projects, including billions of dollars for NBS to fortify coastal communities and improve resilience [40, 90, 91]. In Europe, the European Commission (EC) has also allocated funding to advance the development of NBS, including in coastal settings, and mainstream it internationally through the Horizon Europe research program (previously Horizon 2020) [22–24]. Some European countries also have their own national plans for NBS research and development. In Germany, the Climate and Transformation Fund will supply EU €4 billion until 2026, with the goal of improving ecosystem health and resilience [26]. NBS funding and initiatives are also prevalent in Latin American and Caribbean countries, including Mexico and Colombia [64] and Asian countries, including China [10] and Japan [82].

Despite recent increases in global implementation of NBS projects for coastal protection, substantial gaps in our understanding of NBS performance exist both broadly [73] and relative to coastal protection [70]. These gaps proliferate due to a lack of studies on the broader effectiveness of NBS, especially in coastal areas; a recent review of NBS effectiveness found that only 13% of studies were conducted in coastal ecosystems, including coral reefs, mangroves, seagrass communities, and salt marshes [7]. Most NBS studies do not report on the full suite of NBS performance outcomes [7] because it is challenging to develop, as well as costly to measure, appropriate social and ecological [70, 73], as well as physical and economic [7, 73] performance standards. For example, measuring cost-effectiveness of NBS is difficult because the protection NBS affords depends on a variety of factors, such as the intensity and frequency of events an area experiences [73] or the time horizon over which costs are considered [25]. This is also the case, however, for gray infrastructure, but a key difference between NBS and gray infrastructure is that NBS protective services are hypothesized to increase over time, while gray infrastructure protective services may decline [25]. NBS assessments are also challenging because performance is strongly influenced by the detailed and often unique place-based context of each project (e.g., geomorphology) [7]; this is also true of gray infrastructure, but many modeling tools and design standards exist to help engineers design structures for specific levels of protection. Many NBS projects do not include budgets or requirements for monitoring, especially long-term monitoring, to ensure that projects meet expectations [32, 51, 61], reinforcing knowledge gaps. Inability to address these gaps in the near future will likely hinder further investment and implementation of NBS [7], including NBS for coastal protection.

Surges in funding and subsequent construction of NBS for coastal protection, combined with the lack of NBS performance knowledge across geographies and conditions, have escalated the need to assess the performance of NBS for coastal protection. This study aims to identify, collate, and map the global evidence base on the ecological, physical, social, and economic performance of active NBS interventions used within the context of coastal protection in six biogenic, shallow (intertidal or subtidal) coastal ecosystems that face a variety of stressors and are among the most imperiled ecosystems on earth [33, 43]. The coastal ecosystems that we selected for inclusion in the systematic map are salt marsh, seagrass, kelp, mangrove, shellfish reef, and coral reef systems. The systematic map scope includes active NBS interventions for coastal protection, such as restoring or creating habitat, adding structure, or modifying sediment or morphology. The decisions to narrow the focus to six coastal ecosystems and active NBS interventions for coastal protection were made based on the primary research and management expertise of the systematic map team, as well as resource constraints. An improved understanding of NBS performance in shallow, biogenic coastal areas will help determine the breadth and depth of the knowledge base, highlighting both knowledge clusters and knowledge gaps.

### Stakeholder engagement

This systematic map was initiated by the National Oceanic and Atmospheric Administration (NOAA) National Centers for Coastal Ocean Science (NCCOS) to determine the state of knowledge regarding the performance of NBS for coastal resilience. The synthesis was motivated by a federally identified need to understand the evidence base surrounding NBS performance to help inform policy and management decisions about how to monitor NBS and when and where to implement NBS, as well as to identify where additional performance evaluations are warranted. Federal “team leads” for the synthesis effort developed a “core team” of federal researchers and academic scientists who study and implement NBS in estuarine and marine ecosystems. The core team helped refine the protocol scope, including research questions, inclusion criteria, and search strategy, and will continue to play key roles in compiling the map. We also convened an “advisory team” of additional scientists and managers with expertise in NBS and coastal ecosystems to provide additional direction and feedback. The advisory team includes scientists and managers from federal agencies, non-profits, and academia in the US. We engaged with the advisory team in one-on-one or small group virtual meetings and discussions. Several members of the advisory team helped refine the protocol by, for example, helping to represent the needs of their sectors, such as coastal managers. Discussions with the advisory team also helped refine our definitions for NBS and coastal protection, intervention typologies, outcome typologies, and data coding approach. The advisory team will remain engaged in map development through activities such as recommending additional sources of evidence to include in the map. As neither our advisory group nor our core team include international scientists, we plan to consult additional scientists from countries outside of the US during map development to help ensure that relevant international literature is incorporated into the map and to reduce bias.

## Objective of the systematic map

The objective of this systematic map is to identify, collate, and map the global evidence base on the ecological, physical, social, and economic performance of active NBS interventions related to coastal protection in salt marsh, seagrass, kelp, mangrove, coral reef, and shellfish reef systems. We use the term “active intervention” to mean the action of intentionally using, constructing, introducing, installing, or implementing NBS. We use the term NBS below to describe NBS for coastal protection rather than NBS more broadly. As such, this systematic map focuses on biogenic coastal ecosystems with active NBS interventions for coastal protection rather than conservation of existing, relatively intact ecosystems and the coastal protective services they provide.

Question: What is the extent and distribution of evidence on the ecological, physical, social, and economic performance of active NBS interventions used in salt marsh, seagrass, kelp, mangrove, coral reef, and shellfish reef systems within the context of coastal protection?

Sub-questions: We define performance as the suite of evaluated ecological, physical, economic, or social outcomes from active NBS interventions in six coastal ecosystems. We ask the following sub-questions about NBS performance:

- Which coastal protection services (e.g., reduce shoreline erosion, attenuate wave energy, reduce inundation) do active NBS interventions seek to deliver?
- How does the extent and distribution of evidence on NBS performance differ across ecological (e.g., species and population, biological interactions, nutrient cycling), physical (e.g., water level, waves, sediment and morphology), social (e.g., human health, culture, safety and security), and economic (e.g., income, livelihoods, natural capital) outcomes?
- How does the extent and distribution of evidence on NBS performance differ by ecosystem type (e.g., salt marsh, mangrove, shellfish reef), NBS intervention type (e.g., system restoration or enhancement, system creation, structure addition), geographic location, and spatial scale?
- What approaches or methods are used to assess NBS performance? When is performance assessed relative to NBS implementation (e.g., < 1 yr, 1–5 yrs, 5–10 yrs, > 10 yrs after construction)? What comparative approaches, if any, are used to assess NBS performance (e.g., presence vs. absence of NBS intervention, different types of NBS interventions, natural system vs. NBS intervention, no comparator)?
- Which metrics (e.g., aboveground biomass, job creation) are used to assess NBS performance?

Elements of the primary question: Elements of the primary question include the population, intervention, comparator, outcome, and study type (Table 1).

**Table 1:** Summary of elements of the primary question, including population, intervention, comparator, outcome, and study type

Population	Salt marsh, seagrass, kelp, mangrove, shellfish reef, or coral reef systems where active NBS interventions are used
Intervention	Active NBS interventions established within the context of coastal protection
Comparator	No comparator required beyond presence of an active NBS intervention
Outcome	Ecological, physical, economic, or social performance outcomes evaluated following NBS interventions
Study type	Experimental, quasi-experimental, observational, or modeling studies with quantitative or qualitative data on NBS performance outcomes

## Methods

The systematic map will adhere to the Collaboration of Environmental Evidence (CEE) Evidence Guidelines and Standards for Evidence Synthesis [12] and conform to the RepOrting standards for Systematic Evidence Synthesis (ROSES) for systematic map protocols [42] (Additional file 1).

### Search strategy

A comprehensive search will be performed to acquire traditional peer-reviewed publications and gray literature using bibliographic databases, indexing platforms, open discovery citation indexes, a novel co-citation and bibliographic coupling literature discovery tool, a web-based search engine, and organizational databases and websites. Our strategy will also include hand-searching reference sections of relevant reviews found during initial scoping to identify publications that may not be found in our search. Finally, we will engage with stakeholders to identify additional publications that may not be discovered in our search.

All searches will be performed from 1980 to present. This temporal scope is based upon a review of living shorelines, a common type of NBS, in which the earliest known study uncovered in the scoping review was from 1981 [77], suggesting that most studies on NBS with performance monitoring will be from 1980 to present. We realize that older NBS exist [52]; our temporal scope cutoff of 1980 will not necessarily preclude our search from including performance evaluations of older NBS but will restrict our search to evaluations published in 1980 or after. All searches will be conducted in English, and only studies with English language full text will be included. Since many non-English language articles include English language abstracts, studies included at the title and abstract screening phase but whose full text is not published in English, will be excluded and noted as non-English during full text screening to aid future research that could be completed in additional languages. We acknowledge that limiting the language to English will introduce bias to our search because we may exclude relevant articles solely on the basis that they are not written in English. Despite the English-language focus of our search, the systematic map will include global evidence, regardless of country of origin, so long as it is available in English, because it is useful to evaluate global evidence across ecosystems and jurisdictional boundaries. Some countries are at different stages of designing, implementing, and evaluating NBS for coastal protection, and so the global scope will help catalog the English-language evidence base rather than evidence from one or several countries. We recognize, however, that decisions on how to design, site, and implement NBS are often location-specific and that the systematic map will not provide localized information, beyond capturing individual location-specific studies, but rather a broader knowledge base upon which to build in the future. Subscriptions from the NOAA Central Library and Duke University will be used to access databases and platforms that are not publicly available.

#### Keyword development

Initial keywords related to the elements of the primary question for NBS (intervention), coastal ecosystems (population), and coastal protection (intervention) were developed by a team of subject matter experts and librarians. Additional keywords for each topic were then identified for testing and review from known review articles and an initial set of benchmarking articles. Next, further keywords were developed by text mining, in which terms were reproducibly selected from a sample set of literature using the R package ‘litsearchr’ [35, 36]. Once keywords were extracted for NBS, coastal ecosystems, and coastal protection, subject matter experts and librarians reviewed these keyword lists and selected keywords for further testing during the search string development phase.

An example of this process for NBS keyword development is as follows. Keywords related to the broad concept of NBS and similar concepts such as nature-based infrastructure (NBI), natural and nature-based features (NNBF), and green infrastructure were developed by a team of subject matter experts and librarians. A simple search string was created and applied in Web of Science to test the keywords and capture a focused set of relevant literature:

(TI=(“nature based solution*” OR “nature based infrastructure” OR “living shoreline*” AND “coastal protection”)) OR AB=(“nature based solution*” OR “nature based infrastructure” OR “living shoreline*” AND “coastal protection”).

In this search, the (*) is a wildcard, which represents any character, including no character. Quotation marks are used to search exact phrases. Due to the mechanics of Web of Science, the search “nature based solution*” includes variations such as “nature-based solution,” “nature based solutions,” and “nature-based solutions.” Following this simple search, results were exported and run through the R package ‘litsearchr,’ which uses text-mining and keyword co-occurrence to identify potential keywords in a reproducible, quasi-automated method [35, 36]. The package allows users to adjust both the minimum frequency (wherein a keyword must be discovered in a set number of sources) and n-gram length (a contiguous sequence of n items). The ability to extract keywords and phrases sped up our process of keyword building and provided our team with a more comprehensive list of keywords for review and testing. Resulting keywords were then reviewed by subject matter experts and librarians and used to build search strings. This process was repeated for elements of the primary question related to coastal ecosystems and coastal protection.

To complement the keyword development approach detailed above, we reviewed strings from previously published studies to generate additional keywords. Specifically, we reviewed the search string used in a scoping review of living shorelines [77], a systematic map from the UK on how NBS contributes to human well-being [16], and a systematic map protocol on natural climate solutions and mitigation outcomes [9]. Relevant terms that we had not yet identified from these three article’s search strings were added to our list of keywords. We also used ‘litsearchr’ to text-mine titles and abstracts included in the living shoreline scoping review [77] to develop additional keywords.

#### Search string development

Using the compiled keyword lists, search strings were developed to align with the key elements of the primary question representing the population and interventions (Tables 1, 2, Additional file 2). The population search string targeted eligible coastal ecosystems (i.e., salt marsh, shellfish reef, coral reef, mangrove, seagrass, kelp) and also included more general terms, like estuary and vegetation, used to refer to these ecosystems (Table 2, Additional file 2). The intervention search string was more complex because of the difficulty of searching for articles that reported on NBS intended to mitigate against coastal hazards and provide coastal protection benefits. We developed three substrings for the intervention string: (1) NBS, (2) hazards, and (3) mitigation (Table 2, Additional file 2). Both hazards and mitigation help identify papers focused on coastal protection. We did not develop a search string for outcomes because we wanted to cast a broad net across the range of possible outcomes in ecological, physical, social, and economic areas. Web of Science Core Collection was used to develop and test all search strings. The search string development process and associated decisions are documented in Additional file 2.

**Table 2:** Search substrings created for population and interventions

PIO criteria	Concept	Substring (Web of Science syntax)
Population	Coastal ecosystems	(TI=(oyster* OR mussel* OR bivalve* OR shell* OR cultch* OR coral* OR reef* OR marsh* OR saltmarsh* OR wetland* OR estuar* OR kelp OR seaweed* OR seagrass* OR "sea grass*" OR mangrove* OR swamp* OR mangal* OR "aquatic plant*" OR vegetation) OR AB=(oyster* OR mussel* OR bivalve* OR shell* OR cultch* OR coral* OR reef* OR marsh* OR saltmarsh* OR wetland* OR estuar* OR kelp OR seaweed* OR seagrass* OR "sea grass*" OR mangrove* OR swamp* OR mangal* OR "aquatic plant*" OR vegetation))
Intervention	NBS	(TI=("nature based solution*" OR "nature based strateg*" OR "nature based defen$e*" OR "nature based protection*" OR "nature based coastal" OR "nature based shoreline*" OR "nature based mitigation" OR "nature based infrastructure" OR "hybrid infrastructure" OR "hybrid technique*" OR "natural climate solution*" OR "natural infrastructure" OR "eco* engineer*" OR "ecosystem friendly engineering" OR bioengineer* OR "blue engineering" OR "building with nature" OR "engineering with nature" OR "working with nature" OR "nature derived solution*" OR "nature based feature*" OR "nature inspired solution*" OR "nature inclusive design*" OR "nature inspired design*" OR "nature derived design*" OR "soft protection strateg*" OR "soft shoreline*" OR "coastal adaptation*" OR "ecosystem* based adaptation*" OR "ecosystem* based measure*" OR "ecosystem* based mitigation" OR "disaster risk reduction" OR "living shoreline*" OR "coastal defen$e*" OR "natural barrier*" OR bioshield* OR "coastal protection" OR "protect* coast*" OR "shoreline protection*" OR "blue infrastructure" OR "soft defen$e*" OR "shoreline defen$e*" OR "managed realignment" OR "ecosystem based disaster risk reduction" OR "coastal resilienc*" OR "shoreline resilienc*" OR "restor* ecosystem* function*") OR AB=("nature based solution*" OR "nature based strateg*" OR "nature based defen$e*" OR "nature based protection*" OR "nature based coastal" OR "nature based shoreline*" OR "nature based mitigation" OR "nature based infrastructure" OR "hybrid infrastructure" OR "hybrid technique*" OR "natural climate solution*" OR "natural infrastructure" OR "eco* engineer*" OR "ecosystem friendly engineering" OR bioengineer* OR "blue engineering" OR "building with nature" OR "engineering with nature" OR "working with nature" OR "nature derived solution*" OR "nature based feature*" OR "nature inspired solution*" OR "nature inclusive design*" OR "nature inspired design*" OR "nature derived design*" OR "soft protection strateg*" OR "soft shoreline*" OR "coastal adaptation*" OR "ecosystem* based adaptation*" OR "ecosystem* based measure*" OR "ecosystem* based mitigation" OR "disaster risk reduction" OR "living shoreline*" OR "coastal defen$e*" OR "natural barrier*" OR bioshield* OR "coastal protection" OR "protect* coast*" OR "shoreline protection*" OR "blue infrastructure" OR "soft defen$e*" OR "shoreline defen$e*" OR "managed realignment" OR "ecosystem based disaster risk reduction" OR "coastal resilienc*" OR "shoreline resilienc*" OR "restor* ecosystem* function*"))
Intervention	Hazards (coastal protection)	(TI=("coastal hazard*" OR "extreme weather" OR "extreme event*" OR "severe storm*" OR tsunami* OR typhoon* OR cyclon* OR hurricane* OR "tropical storm*" OR "storm surge*" OR monsoon* OR northeaster* OR nor'easter OR "sea level*" OR "high wind" OR "wave action") OR AB=("coastal hazard*" OR "extreme weather" OR "extreme event*" OR "severe storm*" OR tsunami* OR typhoon* OR cyclone* OR hurricane* OR "tropical storm*" OR "storm surge*" OR monsoon* OR northeaster* OR nor'easter OR "sea level*" OR "high wind" OR "wave action")) AND (TI=(erosion OR erod* OR flood* OR inundat* OR "storm surge*") OR AB=(erosion OR erod* OR flood* OR inundat* OR "storm surge*")) AND (TI=(coast* OR shoreline* OR *tidal OR estuar* OR marsh*) OR AB=(coast* OR shoreline* OR intertidal OR subtidal OR tidal OR estuar* OR marsh*))
Intervention	Mitigation (coastal protection)	(TI=(reduc* OR mitigat* OR protect* OR dissipat* OR dampen* OR attenuat* OR stabili$* OR trap* OR buffer* OR armour* OR armor* OR barrier* OR accret* OR adapt* OR breakwater*) OR AB=(reduc* OR mitigat* OR protect* OR dissipat* OR dampen* OR attenuat* OR stabiliz* OR trap* OR buffer* OR armour* OR armor* OR barrier* OR accret* OR adapt* OR breakwater*)) AND (TI=(hazard* OR erosion OR erod* OR flood* OR "storm surge*" OR wave* OR soil OR sediment* OR substrat* OR shoreline*) OR AB=(hazard* OR erosion OR erod* OR flood* OR "storm surge*" OR wave* OR soil OR sediment* OR substrat* OR shoreline*)) AND (TI=(coast* OR shoreline* OR *tidal OR estuar* OR marsh*) OR AB=(coast* OR shoreline* OR intertidal OR subtidal OR tidal OR estuar* OR marsh*))
Intervention	Restoration	(TI=(construct* OR plant* OR install* OR restor* OR enhance* OR creat* OR retrofit*) OR AB=(construct* OR plant* OR install* OR restor* OR enhance* OR creat* OR retrofit*))

Substrings are in Web of Science Syntax, where “TI” indicates title and “AB” indicates abstract

The population and intervention search strings (Table 2) were employed together in different combinations to capture particular types of articles (Table 3). For example, we combined strings for populations and NBS to search for articles where NBS has been used in the six target coastal ecosystems regardless of whether coastal protection is referenced in the title or abstract. We then created a string combining NBS and hazards and one with NBS and mitigation to find articles where coastal NBS has been used either in reference to hazards or specifically to mitigate hazards, respectively. Some articles of interest do not explicitly refer to NBS interventions using NBS or related terms like green infrastructure, so we designed another search string combination of population and both coastal hazards and mitigation to find these relevant articles. Other articles use mitigation language and restoration language (e.g., restoration, mitigation, enhancement) but do not explicitly use NBS terms or hazards, so we created a final search string combination to detect these articles.

**Table 3:** Search string combinations employed to capture articles on NBS intended for coastal protection

String combination	Search designed for
NBS AND population	Articles focused on NBS concepts from target coastal ecosystems
NBS AND mitigation	Articles focused on NBS concepts and coastal mitigation actions that do not explicitly mention target ecosystems in title or abstract
NBS AND hazards	Articles focused on NBS concepts and coastal hazards that do not explicitly mention target ecosystems in title or abstract
Population AND mitigation AND hazards	Articles focused on coastal ecosystems and hazards and mitigations that do not explicitly use NBS or related terms in title or abstract
Population AND mitigation AND restoration	Articles focused on coastal ecosystems and mitigations that do not explicitly use NBS or related terms in the title or abstract but do use terms related to habitat restoration and creation

#### Searching the literature

##### Indexing platforms, bibliographic databases, and open discovery citation indexes

Select indexing platforms, bibliographic databases, and open discovery citation indexes will be searched for relevant articles (Table 4). Since search strings were developed based on the syntax used by Web of Science, we will modify search strings as needed to ensure proper source-specific syntax or restrictions. Any variations or modifications to the final search strings will be documented, and any source-specific filters or limiters used to implement searches will be noted to ensure search transparency and reproducibility.

**Table 4:** List of indexing platforms, bibliographic databases, open discovery citation indexes, and the web-based search engine and novel literature discovery tool incorporated into the search strategy

Source type	Source name	Indexes	Subscription	Limits, restrictions, or filters	Platform or provider
Indexing platforms	Scopus	Scopus	Duke University	Year: 1980–present	Elsevier
	Web of Science Core Collection (WoS)	SCI-expanded (1980–present) SSCI (1980–present) CPCI-S (1990–present) CPCI-SSH (1990–present) ESCI (2018–present)	Duke University	Year: 1980–present Document type: article, proceeding paper, early access, data paper	Clarivate
Bibliographic databases	Ocean abstracts (1981–present)	N/A	NOAA	Year: 1980–present Source type: scholarly journals, dissertations & theses, conference papers & proceedings, reports	ProQuest
	Earth, atmospheric, & aquatic sciences collection	Databases included Aquatic sciences and fisheries abstracts Meteorological and geoastrophysical abstracts Earth, atmospheric, & aquatic sciences database	NOAA	Year: 1980–present Source type: scholarly journals, dissertations & theses, conference papers & proceedings, reports	ProQuest
Open discovery citation indexes	LENS.org	CORE Crossref PubMed Microsoft Academic	N/A	Year: 1980–present Document type: journal article, conference proceeding article, conference proceedings, dissertation, report	Cambia
	Dimensions	N/A	NOAA	Year: 1980–present Publication type: article, proceeding	Digital science
Web-based search engine	Google Scholar	Google Scholar	N/A	Title search Up to the first 1000 results	Google Scholar via Publish or Perish [44]
Novel literature discovery tool	Inciteful	N/A	N/A	Up to the first 1000 results	[89] (https://inciteful.xyz/)

For each source, the indexes, subscription, and provider are provided. Limits, restrictions, or filters are also noted

##### Web-based search engine

Google Scholar will be searched for relevant articles (Table 4). Given that Google Scholar has reduced capabilities to implement Boolean logic compared to platforms like Web of Science [41], we will adapt our search string for Google Scholar using the most relevant search string components. We will perform the search on article titles because title searches tend to return more gray literature than full text searches [41]. The search will be implemented via Publish or Perish software [44] to ensure that relevant articles can be exported as a .RIS file. We will screen the first 1000 search returns from Google Scholar. We selected this number of search returns based on recommendations for searching Google Scholar peer-reviewed literature and gray literature for systematic reviews [41].

##### Novel literature discovery tool

The novel literature discovery tool “Inciteful” [89] will be used to search for additional literature (Table 4). Inciteful is an online tool that allows articles to be uploaded (.BIB file) and then provides a list of similar papers. We will seed the tool using select benchmarking articles. We will export up to 1000 most similar articles.

##### Organizational databases and websites

Forty-four organizational databases and websites (Table 5) will be searched for relevant gray literature not reflected in indexing platforms, bibliographic databases, open discovery citation indexes, novel literature discovery tools, and web-based search engines. The organizations include governmental organizations, non-profit organizations, and academic institutions that fund, implement, or monitor NBS in coastal systems. Organizational databases (e.g., repositories) contain searchable collections of literature produced by, associated with, or funded by a particular organization (e.g., NOAA institutional repository). Organizational websites include those that contain NBS performance evidence but within a less formal framework than a database or repository, such as a list of publications on NBS performance evaluations.

**Table 5:** Organizations whose databases and websites will be searched for evidence on NBS performance

Organization name	URL
Asian Development Bank	https://www.adb.org/
Australian Government Department of Climate Change, Energy, the Environment, and Water	https://www.dcceew.gov.au/
Billion Oyster Project	https://www.billionoysterproject.org/
Caribbean Natural Resources Institute	https://hub.canari.org/
Climate Resilient by Nature	https://www.climateresilientbynature.com/
ClimateLinks	https://www.climatelinks.org/
Commonwealth Scientific and Industrial Research Organisation	https://www.csiro.au/
Conservation International	https://www.conservation.org/
UK Government Department for International Development	https://www.gov.uk/
USAID Development Experience Clearinghouse	https://www.usaid.gov/
Duestsche Gesellschaft fur Internationale Zusammenarbeit	https://www.giz.de/
Environmental and Energy Study Institute	https://www.eesi.org/
Environmental Defense Fund	https://www.edf.org/
European Union/Commission	https://op.europa.eu/
Global Facility for Disaster Reduction and Recovery	https://www.gfdrr.org/
Global Mangrove Alliance	https://www.mangrovealliance.org/
Global Program on Nature-Based Solutions for Climate Resilience	https://naturebasedsolutions.org/
iied Publications Library	https://www.iied.org/
International Monetary Fund	https://www.imf.org/
International Union for Conservation of Nature	https://www.iucn.org/
National Fish and Wildlife Foundation	https://www.nfwf.org/
National Oceanic and Atmospheric Administration	https://www.noaa.gov/
National Science Foundation	https://www.nsf.gov/
Oxford Nature Based Solutions Initiative	https://www.naturebasedsolutionsinitiative.org/
Rare	https://rare.org/
Resources for the Future	https://www.rff.org/
The Nature Conservancy	https://www.nature.org/
United Nations Decade on Restoration	https://www.decadeonrestoration.org/
United Nations Development Programme	https://www.undp.org/
United Nations Environment Programme	https://www.unep.org/
United Nations Environment Programme World Conservation Monitoring Center	https://resources.unep-wcmc.org/
United States Army Corps of Engineers	https://www.usace.army.mil/
United States Climate Resilience Toolkit	https://toolkit.climate.gov/
United States Department of Transportation	https://www.transportation.gov/
United States Environmental Protection Agency	https://www.epa.gov/
United States Fish and Wildlife Service	https://www.fws.gov/
United States Geological Survey	https://www.usgs.gov/
University of Georgia Institute for Resilient Infrastructure Systems	https://iris.uga.edu/
Wetlands International	https://www.wetlands.org/
Wildlife Conservation Society	https://library.wcs.org/
World Agroforestry Center	https://www.worldagroforestry.org/
World Bank	https://www.worldbank.org/
World Resources Institute	https://www.wri.org/
World Wildlife Fund	https://www.worldwildlife.org/

The name of each organization and the URL are provided

Most organizational databases and websites do not allow Boolean searches so the detailed search strings (Table 2) will be adapted for “by hand” searches. The search string used may vary by database or website but will include a keyword or combinations of keywords, or a built-in website filtering function (e.g., dropdown menu to filter by document category or topic). Some websites, however, do not have search functions so must be searched manually. For each organizational database or website, the first 100 search results will be screened in situ*.* Relevant gray literature discovered from these sources will be added to the systematic map database, but articles screened in situ as not relevant will be excluded and thus not added to the database. We will record the website name, URL, date searched, search method (filtered, keyword, search string, by hand), and number of relevant articles identified for each organizational database or website.

### Comprehensiveness of the search

The stakeholder team identified 55 relevant articles to test our search string against (Additional file 3). These articles, which we refer to as benchmarking articles, were sourced from subject matter experts. Some benchmarking articles were also sourced from Smith et al. [77], a recent scoping review of living shorelines. The identified benchmarking articles met the eligibility criteria and would be included at the full text stage. We implemented our search string in the Web of Science Core Collection and tested whether our benchmarking articles were returned by our search strings. Of the 55 benchmarking articles 52 were indexed in Web of Science. Our initial search results failed to identify nine (6 indexed, plus 3 not indexed) of the benchmarking articles. We then adjusted our search string incrementally until it captured all 52 benchmarking articles indexed in Web of Science Core Collection; in total, we conducted five rounds of testing search string variations, improving searches, and refining combinations of substrings during the benchmarking stage. We used functionalities within the R package ‘CiteSource’ [69] to evaluate how changes to the search string affected the number of found benchmarking articles. We also used ‘CiteSource’ to identify which search strings and combinations found unique benchmarking articles, versus which were duplicative, and which increased or reduced search precision. We verified that the three articles not indexed in Web of Science were returned in searches via open discovery citation indexes like LENS and Dimensions. Following benchmarking, research librarians and subject matter experts peer-reviewed the search strings and strategy to ensure consistent use of syntax like truncations, and the search strings were updated based on reviewer feedback.

### Reference management and deduplication

All references will be managed using Clarviate’s EndNote (version 20 and version 9) citation management software [81]. Search results from indexing platforms, bibliographic databases, open discovery citation indexes, Google Scholar via Harzings, and the novel literature discovery tool will be exported as separate .RIS files and imported into EndNote. References within each .RIS file will be deduplicated using ‘CiteSource,’ which deduplicates within a database (e.g., WoS) and then across databases (e.g., WoS, LENS, etc.). A combined deduplicated .RIS file will be exported from CiteSource and imported to EndNote. Within EndNote, we will perform manual deduplication to identify any citations that could not be merged via automated deduplication in CiteSource. We will use the built-in deduplication function within EndNote, to find and analyze any citations with matching combinations of (1) DOI, (2) title and author, and (3) author and year. Metadata quality will be checked within EndNote to ensure completeness among metadata fields and, specifically, that both title and abstract are available. Search results from the different platforms will be combined into a single .RIS file and imported into Swift Active Screener [45] along with benchmarking or seed articles. Screening of titles and abstracts will be conducted in Swift, and when screening is complete, we will export .RIS files of all included articles, all excluded articles, as well as other Swift-generated reports. The .RIS file from Swift will be imported into EndNote, where—in preparation for full text screening—we will add full texts of articles that passed inclusion criteria and finalize metadata (e.g., year, title, DOI). We will keep a record of articles for which we could not locate full texts.

During full text screening, screeners will simultaneously operate EndNote and Google Sheets. They will use EndNote to screen the full texts stored within EndNote for each article and will highlight salient portions of each article that relate to eligibility criteria or other metadata attributes. Screeners will code metadata attributes in Google Sheets during full text screening. Google Sheets provides an open access spreadsheet that can be used simultaneously by multiple users across institutions and can be populated with dropdown options. More specifically, each row of the Google Sheet will correspond to an article requiring full text screening. Initial columns of the Google Sheet corresponding to metadata fields like title, authors, and publication date will be populated from a .RIS file exported from EndNote converted to a.CSV file and fed into Google Sheets.

The subset of studies that pass full text screening will be combined into a single .RIS file. All .RIS files from title and abstract screening, full text screening, and the final included articles will be available as additional files or archived as part of the systematic map.

## Article screening and study eligibility criteria

### Screening process

Articles discovered during the search process will be screened at the level of title and abstract to determine whether they meet predefined inclusion criteria (Table 6). Screening at the title and abstract level will be conducted in Swift Active Screener [45], which is a reference screening software application designed for systematic reviews. Swift Active Screener uses a type of machine learning called active learning. Specifically, it employs active learning to rank publications in order of relevance based on screener feedback so that relevant publications can be screened earlier rather than later. The software updates the order and relevance of publications based on completed reference screening actions. The software also presents a running estimate of the percentage of relevant references that have been screened from the initial set, referred to here as the ‘recall rate’, and a running estimate of the number of remaining relevant references that have not been screened. Those estimates allow a user to define a target recall rate at which point the screening is terminated. We selected a target recall rate of 95% at which point we will terminate further screening [45]. Swift Active Screener has been demonstrated to save significant time resources through its active learning algorithm and associated ranking system. For example, an analysis of datasets used for 26 systematic reviews found that with a 95% recall rate, the median true recall rate using the software was 99% [45]. Multiple systematic review protocols (e.g., [15, 28]) from the medical sciences, which similar to environmental sciences are also held to very high standards, have been published using Swift Active Screener; however, Swift Active Screener has not been used frequently within environmental sciences. We recognize that using Swift Active Screener may introduce bias into our map results because multiple articles could be ranked low and thus targeted for exclusion that might actually warrant inclusion and so could be overlooked. However, based on Howard et al.’s [45] analysis, we think using Swift Active Screener for this systematic map where we expect over 30,000 articles requiring screening is necessary and beneficial.

**Table 6:** Summary of preliminary inclusion and exclusion criteria for literature on NBS performance for coastal protection

Criteria	Overview	Included	Excluded
Population of subjects	Coastal ecosystems with NBS	Salt marsh, seagrass, kelp, mangrove, shellfish reef, or coral reef systems where NBS interventions are used Notes: If one of these six systems is created by an NBS intervention, it is also eligible (e.g., NBS intervention established salt marsh in former mudflat) Salt marsh is defined as estuarine or brackish marsh (not freshwater) Shellfish reefs are defined as oyster, mussel, or other reef-forming bivalves Coral reefs are defined as shallow systems (not deep water or mesophotic) Artificial structures will be included if they fit the population criteria and have been installed in one of the six coastal ecosystems or have a goal of restoring one of the six coastal ecosystems	Terrestrial, freshwater, subterranean ecosystems. Coastal, or marine ecosystems (e.g., rocky reef, dune, beach, maritime forest, deep coral) that are not salt marsh, seagrass, kelp, mangrove, shellfish reef, or coral reef. Freshwater marshes, non-reef building bivalves, are also excluded
Intervention	Active NBS intervention related to coastal protection	Active NBS interventions used within the context of coastal protection. These actions generally involve working with nature to address societal challenges, providing benefits for human well-being, ecosystem services, resilience, and biodiversity [72, 85] Interventions must be an **active NBS intervention** that is used, installed, constructed, or implemented by humans. Active interventions include the following (Table 7): Restore, create, enhance, or rehabilitate natural habitat, ecosystems, or associated services Create habitat or ecosystem in place of a naturally occurring one Add artificial or engineered structure of human origin, natural origin, or hybrid origin to an existing ecosystem Retrofit, modify, or remove gray infrastructure Stabilize, remove, or place sediment in an ecosystem Modify morphology of an ecosystem Remove or add invasive species to an ecosystem Interventions must be related to **coastal protection** NBS stated to have goal, aim, or intent of coastal protection NBS evaluated for coastal protection physical outcomes of any directionality	Passive NBS interventions, as well as active interventions that are not related to coastal protection, are excluded Passive NBS interventions are excluded: NBS that involve protecting, conserving, or managing coastal ecosystems rather than actively restoring, creating, adding structure to, or otherwise modifying ecosystems NBS interventions that have been designed, planned, or sited but not implemented Existing ecosystems without NBS intervention (e.g., a salt marsh that inherently provides coastal protection and other benefits but does not have an active intervention like restoration or structure addition) Active NBS intervention unrelated to coastal protection (e.g., oyster reef restoration) that is for fishery enhancement) are excluded
Comparator	NBS performance	No comparator is required for the systematic map because the only requirement is the presence of NBS intervention, which is not a comparator Studies that include a comparator will also be included. These comparators can be either temporal or spatial Temporal comparators include those that report NBS performance over time gleaned from long-term monitoring, experimental observations, or before vs. after NBS intervention Spatial comparators include those that report NBS performance over space gleaned from locations with or without NBS interventions, or locations with different types of NBS interventions	N/A—no comparator required
Outcome	NBS performance outcomes	Ecological, physical, economic, or social performance outcomes of NBS that are measured, observed, or modeled	Performance outcomes of NBS that **do not** fall within ecological, physical, social, and/or economic categories
Study type	Experimental, observational, or modeling studies	Experimental, quasi-experimental, modeling, or observational (e.g., monitoring or assessment) studies with quantitative or qualitative data	Theoretical studies, commentaries, editorials, opinions, or perspectives

Bold values correspond to key aspects of the inclusion criteria

To help facilitate screening within Swift Active Screener, we will manually add keywords into the Swift Active Screener interface so that they are highlighted in titles and abstracts. We will also add questions for each article that screeners will answer (check boxes, select one or select multiple) within the software application to record whether the article should be included or excluded based on eligibility criteria. We will maintain a full list of excluded articles. In cases where it is unclear whether the article meets screening criteria based on information contained within the title and abstract, the article will be included at the title and abstract screening stage and subjected to further screening at the full text level.

Articles that are deemed to meet inclusion criteria during title and abstract screening will then be screened at the full text level using the same inclusion criteria. If a full text for an article cannot be obtained using all available resources, the article will be excluded. If an article does not meet inclusion criteria during full text screening, it will be excluded. We will maintain a list of studies excluded at the full text screening stage and the reason for exclusion.

To reduce bias during screening, we will hold two training sessions—one for title and abstract screening and one for full text screening—for all screeners to attend. During the training sessions, we will collaboratively work through screening several articles. We will then assign each screener the same small subset of articles to screen. We will compare screening outcomes, discuss inconsistencies, and may alter eligibility criteria if needed. We will evaluate inter-reviewer consistency for the final training set of articles at the title and abstract stage using the Kappa statistic. Given the high number of expected articles, we will conduct double screening for as many as 5% of articles at the title and abstract or full text screening stages. The exact percentage of articles for which double screening will be conducted will depend on the number of total articles, and we will report this information in the systematic map. We recognize that single screening may introduce bias to the systematic map, but it is necessary because of the high number of expected articles (~ 30,000) and resource constraints. If a screener is an author of an article, they will not be permitted to screen the article at the title and abstract or full text stage nor permitted to code metadata extraction.

### Eligibility criteria

To pass title and abstract and full text screening, articles must meet the following eligibility criteria (Table 6).

#### Relevant population(s)

This systematic map focuses on six types of shallow coastal ecosystems: salt marsh, seagrass, kelp, mangrove, shellfish reef, and coral reef. These systems can be either existing (e.g., where NBS is constructed in an existing salt marsh or near an existing salt marsh) or created (e.g., NBS constructed to create salt marsh in an area where salt marsh is currently nonexistent) (Table 6). The six ecosystem types were selected because they are biogenic (e.g., habitat formed by flora or fauna), characterized as intertidal or subtidal, and are increasingly susceptible to coastal development [33] and other human-induced stressors [43]. Other coastal systems, such as dunes, beaches, rocky reefs, and maritime forests, were excluded because, even though they can host active NBS interventions, these systems were deemed beyond the scope of the study by the evidence map team based on time and resource constraints. If, however, a study includes one or more of the six eligible ecosystems and one or more of the excluded ecosystems, the study would be included. For instance, if a study reports on kelp and rocky reefs, the study would be included since it reports on one of the six target ecosystems, even though it also includes content on an excluded system. The included systems provide a range of latitudinal case studies. For instance, some systems are constrained to tropical (coral reefs) or temperate (kelp) latitudes, whereas others are widespread across latitudinal gradients (shellfish reefs). Deep sea, freshwater, subterranean, and terrestrial systems fell outside of the scope of this systematic map.

#### Relevant intervention(s)

A diversity of NBS types are used in coastal ecosystems to solve problems ranging from biodiversity loss and habitat degradation to pollution and coastal development. We scoped this systematic map to focus on a subset of active NBS interventions related to coastal protection (Table 6). To be active interventions, NBS must be used, installed, constructed, or implemented by humans, such as through actions like restoring or creating habitat, adding structure, or modifying sediment or morphology. To be related to coastal protection, NBS interventions must either have a stated goal or evaluated outcome of coastal protection. To meet the “stated goal” provision, NBS must be stated to have a goal, aim, or intent of coastal protection related to waves, current, wind, water level, storm surge, sediment, or morphology. To meet the evaluated outcome provision, NBS must be evaluated for physical outcomes (any directionality—positive, negative, neutral) related to waves, current, wind, water level, storm surge, sediment, or morphology.

NBS for coastal protection range from green (e.g., marsh replanting to reduce coastal erosion) to hybrid (e.g., construction of rock breakwaters with marsh replanting) to gray (e.g., eco-concrete) [4, 25], and we created a typology to encompass these diverse NBS interventions (Table 7; Additional file 4). We include fully green and hybrid active NBS interventions related to coastal protection. We also include gray or engineered structures that have incorporated nature-inspired [48] or nature-derived [48] designs (e.g., concrete module used to create oyster substrate for wave attenuation), as well as actions retrofitting, modifying, or removing gray infrastructure. Specifically, if a human-made structure (gray, hybrid) is installed in one of the six coastal ecosystems or is installed with a goal of restoring one of the six coastal ecosystems, it will be included so long as it meets the other PIOS criteria.

**Table 7:** Typology of NBS interventions. Typologies adapted from Bridges et al. [4], Bridges et al. [5], Brooks et al. [6], Chausson et al. [7], Cheng et al. [9], IUCN [47], Seddon et al. [73]; see additional details in Additional file 4

Category	Definition	Example
System restoration, enhancement, or rehabilitation	Intervention restoring, enhancing, or rehabilitating natural habitat or systems and associated services	Restoration; habitat enhancement; rehabilitation; repopulation; these are for when a habitat currently exists that is improved upon or expanded; code as creation if it is a new system
System creation	Intervention creating system in place of a naturally occurring one	Creation of salt marsh on urban shoreline; creation of oyster reef on mud flats
Structure addition of human origin (artificial)	Intervention adding artificial, engineered, or designed structure to an existing ecosystem	Reef modules; reef ball; reef dome; mini domes; oyster ball; oyster catcher; oyster castle; biorock; artificial seagrass
Structure addition of natural origin	Intervention adding structure of natural origin (even if from a non-coastal ecosystem) to an existing ecosystem	Rock; oyster cultch; marsh mat; marsh mattress; marsh pillow; marsh blanket; biodegradable element; coir mats; logs; trees; rock sill
Structure addition of hybrid origin (both artificial and natural)	Intervention adding structure of mixed natural and human origin to an existing ecosystem	Gabion basket; shade net; biorock; shell bag; oyster bag; concrete/lattice marsh mattresses
Retrofitting or removing gray infrastructure	Intervention retrofitting, modifying, or removing gray infrastructure	Retrofitting, modifying, or removing: culvert, seawall, bulkhead, berm, dike, levee, tide gate
Sediment stabilization, removal, or placement	Interventions stabilizing, removing, or placing sediment in a system	Sediment stabilization; sediment trapping; sediment or fill removal; beneficial use; beneficial reuse; thin-layer placement or application
Morphology modification	Interventions modifying the morphology of a system through changing the topography or morphology	Grading; regrading; terracing; platforming; island building
Invasive species modification	Interventions involving modification of invasive species through removal or addition	Invasive species removal; invasive species addition

#### Relevant comparator(s)

The systematic map employs a population—intervention—outcome—study type (PIOS) approach and intentionally lacks a formal comparator because any study that includes an active NBS intervention related to coastal protection is included (Table 6). For example, if the ecological performance of the NBS intervention is evaluated at a particular time point or location, that provides evidence on performance outside of a comparator framework. While we will include studies without explicit comparators because they provide valuable “point-based” evidence, we will also include studies with more explicitly identified comparators. These could include temporal (presence vs. absence of NBS, before vs. after NBS, etc.) or spatial comparators (e.g., locations with or without NBS or with different types of NBS).

#### Relevant outcome(s)

This systematic map aims to determine the evidence base surrounding performance of NBS in a variety of coastal systems. We have scoped performance broadly to include four categories: ecological, physical, economic, and social (Table 6). Within each category, we have created typologies to which particular outcomes belong for ecological (e.g., population and species, nutrient cycling; Table 8; Additional file 5), physical (e.g., waves, flooding and inundation; Table 9; Additional file 5), social (e.g., health, culture; Table 10; Additional file 5), and economic (e.g., income, financial capital; Table 11; Additional file 5) outcomes. These typologies will continue to be refined during the screening process. Studies that do not report performance within one of the four main categories (ecological, physical, social, economic) will be excluded because they do not provide evidence for this particular evidence map.

**Table 8:** Typology of ecological outcomes. Typologies are from or adapted from Brooks et al. [6], Cheng et al. [8], O’Leary et al. [62], Reid et al. [68], Smith et al. [77]; see additional details in Additional file 5

Category	Definition	Examples
Population/species	Outcomes focused on characteristics of or changes in species or populations	Abundance; density; biomass; demography (age, size structure); behavior (time spent hiding; time spent feeding; distance from habitat); recruitment; reproduction (fecundity; spawning aggregations, reproductive individuals); species range and spatial extent; dispersal (migration patterns, natal homing, habitat use), connectivity (measured with genetics, microchemistry), body conditions (disease incidence, parasitism rate, toxin level), adaptability, resilience, resistance, or recovery at species level (genetic diversity, heat resistance, salinity tolerance, other stressor tolerance)
Community	Outcomes focused on characteristics of or changes in communities	Community composition and species diversity (abundance, richness, evenness); trophic or food web structure (abundance or diversity of organisms within trophic levels, food web redundancy, number of trophic levels); functional redundancy (degree to which species or groups of species generate similar functions); resilience, resistance, or recovery at community level
Habitat	Outcomes focused on characteristics of or changes in habitats	Habitat quantity (cover and extent—area, volume, height, width, cover; gain or loss in extent); habitat quality (3D complexity, rugosity, fractal dimensions, foundation species density); habitat connectivity or biogeography (degree to which habitats are connected, such as seagrass material in fish gut contents; spillover); upland habitat transition boundary or extent; habitat transgression, migration, or transition
Biological interactions	Outcomes focused on characteristics of or changes in biological or species interactions like facilitation, competition, predation	Competition; predation; mutualism; commensalism; facilitation; herbivory; omnivory; carnivory; zooplanktivory; water filtration (e.g., shellfish filtering water or vegetation slowing water movement); invasive or non-native species interactions with other organisms
Spatial functions and processes	Outcomes focused on characteristics of or changes in spatial ecosystem functions and processes	Spatial distribution, including zonation; connectivity (not at species or habitat level, but rather ecosystem level); dispersal; transgression or migration of ecosystem [space]
Temporal functions and processes	Outcomes focused on characteristics of or changes in temporal ecosystem functions and processes	Succession; colonization; transgression or migration of ecosystem [time]; reaction to pulse or chronic disturbances; resilience, resistance, and recovery
Ecosystem productivity	Outcomes focused on characteristics of or changes in ecosystem productivity	Primary productivity; secondary productivity; energy flow; photosynthesis; respiration; decomposition
Nutrient cycling	Outcomes focused on characteristics of or changes in nutrient cycling	Denitrification; nitrification; carbon sequestration; carbon cycling and storage; phosphorus cycling
Ecosystem health	Outcomes focused on characteristics of or changes in ecosystem health	Turbidity; harmful algal blooms; bacteria, viruses, and fungi; toxins and contaminants; microplastics; debris; bioaccumulation; nutrient levels and pollution; invasive or non-native species ecosystem effects; tipping points and thresholds; resilience, resistance, or recovery at ecosystem level

**Table 9:** Typology of physical outcomes. Typologies are from or adapted from Barbier et al. [2], Bridges et al. [4], Temmerman et al. [80]; see additional details in Additional file 5

Category	Definition	Examples
Waves	Processes or characteristics related to waves, including their energy and height and whether waves are attenuated or amplified	Wave attenuation, dissipation, dampening; wave amplification or build up; wave height; wave length; wave energy; wave breaking; resistance to waves; wave diffraction; wave breaker or breaking; duration; wave exposure; wave orbital velocity; wave runup; wave celerity; wave direction; wave frequency; wave setup; wave steepness
Currents	Processes or characteristics related to currents, including magnitude, direction, and exposure	Speed; direction; exposure; current patterns; circulation
Wind	Processes or characteristics related to wind, including speed, fetch, and buffering	Speed; fetch; direction; buffering; exposure; wind setup; wind stress;
Water level	Processes and characteristics of water level, including flooding and inundation and sea level rise	Flood or inundation height; flood or inundation level; flood or inundation frequency; flood or inundation duration; inundation tolerance; king tide; tides; tidal range; mean sea level; water level
Storm surge	Processes or characteristics related to storm surge, including storm surge height and velocity, as well as storm surge attenuation	Storm surge attenuation or dissipation; storm surge amplification or propagation; storm surge height, elevation, levels, velocity, rates, timing; water storage; resistance to storm surge; surge-attenuation rate
Sediment and morphology	Processes or characteristics related to sediment and morphology, including erosion and accretion and shoreline change	Sediment deposition; sediment accretion; sediment loss; sediment redistribution; sediment compaction; sediment consolidation; elevation buildup; sediment stability; sediment trapping; shoreline change rate; shoreline morphology; shoreline change; elevation change; sediment retention; scour
Sediment and morphology (vertical)	Subset of “sediment and morphology” describing sediment accretion or trapping	Deposition; accretion; redistribution; elevation buildup; trapping
Sediment and morphology (horizontal/lateral)	Subset of “sediment and morphology” describing sediment erosion or shoreline expansion	Lateral erosion; coastal retreat; shoreline recession; resistance to erosion; recession rate; erosion prevention; erosion rate, aggradation; migration; transgression; upland transition; shoreline growth; shoreline expansion

**Table 10:** Typology of social outcomes. Typologies are from or adapted from Brooks et al. [6], Cheng et al. [9], McKinnon et al. [56]; see additional details in Additional file 5

Category	Definition	Examples
Safety and security	Physical security, including personal safety and community sense of safety; vulnerability, resilience, or adaptive capacity	Crime rates; crime severity; acts of violence; threats; emergency services; safety at work; sense of personal security; sense of community security; perceptions of livelihood security in relation change; capacity to deal with shocks; opportunity diversification
Health	Individual mental or physical health or access to health services	Mortality; life expectancy; healthcare access; physical health; emotional health; emotional health; disease occurrence; nutrition; life satisfaction
Education and skills	Formal and informal education and training, capacity building, or associated infrastructure	Formal education; informal skills and training; environmental education; climate change education; literacy rates; school attendance or enrollment; access to education and training; livelihood skills; fellowships; degrees awarded
Knowledge and awareness	Knowledge and awareness of environmental issues and NBS, often as a result of education	Awareness or knowledge of environmental issues, NBS coastal hazards, coastal mitigation, coastal protection, climate change, or related programs; community activities and outreach; awareness building and advertising
Social capital	Social relations, networks, and resources used by individuals and groups to increase well-being, includes external (government) relations	Social cohesion; trust in neighbors; sense of community; community spaces; conflicts; community support; perceptions of discrimination; also external relations between individuals or groups and the government—trust in government ability to resolve conflicts; perception of government efficiency
Culture	Cultural, societal, and traditional values related to nature and natural resources, including community activity and spiritual or religious beliefs	Place attachment; sense of home or place; time spent outdoors; access to nature; outdoor activities; aesthetic value; generational connections and heritage; self-definition at individual and community levels; sense of connection to livelihood; perceptions of one's culture by others; demography of cultural groups (ethnic, religious, linguistic, diversity); use, transfer, or preservation of traditional ecological knowledge and place names; sense of home or place; cultural identity; cultural heritage; spiritual or religious beliefs or values; cultural activities, events, and festivals; cultural practices and customs; traditional use of resources; traditional management activities; social organization; access to sacred sites
Basic infrastructure	Infrastructure necessary for water, sanitation, energy, education, transportation, communication, etc.	Existence, provision, status, or maintenance of basic infrastructure required for individuals and communities, including infrastructure for water, sanitation, energy, education, transportation, communication, etc
Rights, empowerment, and governance	Structures and processes for decision-making, including formal and informal rules; participation or engagement in or control of decision making, accountability, transparency, justice, and empowerment; resource rights	Resource or property rights including withdrawal, exclusion, alienation, management, access; participation in community groups; female participation in user groups; voting rates; financial contribution to community groups or organizations; power or access to power through collaboration or other processes; capacity building initiatives; financial sovereignty; political capital; political organizing; creation, destruction, or changes to institutions for environmental management

**Table 11:** Typology of economic outcomes. Typologies are from or adapted from Cheng et al. [8, 9], Brooks et al. [6], O’Leary et al. [62]; see additional details in Additional file 5

Category	Definition	Examples
Income	Monetary income from wage labor, direct sale of goods, consumption of goods, or value added from natural resources	Individual or household monetary income from wage labor; individual or household monetary income from direct sale of goods (e.g., those derived from natural resources); individual or household physical income from consumption of natural resource goods; individual, household, or community monetary income from value addition from natural resources, coastal protection, nature-based solutions, and/or entrepreneurship; wages; after-tax income rates; income per unit effort
Livelihoods and employment	Occupation, livelihood, and employment and associated opportunities	Employment; employment opportunities; business ownership; employment rates or levels, employment seasonality; employment satisfaction; unemployment trends; job satisfaction and quality; resource-based livelihoods including extractive and non-extractive; non-resource-based livelihoods; sense of employment security; livelihood diversity
Financial capital	Value of financial assets, including those derived from natural resources or NBS	Credit; savings; debt
Natural capital	Stock of natural assets, including those derived from natural resources or NBS an individual has access rights to	Natural resource assets with access or use; salt and exclusion rights; includes coastal habitats, lands, or landscapes
Physical capital	Stock of material assets, including those derived from natural resources or NBS, that an individual has access rights to	Material assets with access or use, including possessions, real estate
Living standards	Living standards of basic life, including economic and material needs	Wealth distribution; poverty indices like unsatisfied basic needs, multidimensional poverty, income vs. expenditure; inflation; shelter or housing availability
Tourism and recreation	Economic value of tourism and recreation opportunities	Subset of several categories above that identifies economic outcomes directly associated with tourism and recreation

#### Relevant study type(s)

Observational (e.g., monitoring, assessment), experimental, modeling/simulation, or quasi-experimental studies will be included from peer-reviewed publications and gray literature (Table 6). These studies will provide data on NBS performance that can be either quantitative measurements or more qualitative comparisons. Theoretical studies will not be included because they are not based on empirical in situ quantitative or qualitative data. Commentaries, perspectives, opinions, and editorials are excluded.

### Study validity assessment

Because we are conducting a systematic map to compile a broad evidence base, we do not plan to systematically assess the study validity through conducting critical appraisals as is typical in systematic reviews. We understand that this may have implications for the utility of the systematic map, such as limiting interpretations surrounding gaps and clusters in evidence. We will acknowledge these limitations in the final map. We will, though, code attributes of each study, such as performance assessment frequency and the method used to evaluate NBS performance outcomes. These attributes can assist end users of the systematic map in making preliminary assessments of study validity.

## Data extraction and coding strategy

Metadata from studies that meet our inclusion criteria will be entered into a standardized data coding spreadsheet (Table 12; Additional file 6). The extracted metadata will include bibliographic (e.g., publication year, authors, title) attributes, as well as attributes describing the population, intervention, study type and—if applicable—the comparator, and outcome. Population metadata attributes will include the ecosystem type and description. Intervention attributes will include the NBS type (Table 6) and description, as well as whether a coastal protection goal accompanies the NBS intervention and if so a description of the goal. Study type attributes will include the type of study (e.g., observational, experimental, modeling), objective, design, geographic location, and comparator. Outcome attributes will include the category and subcategory of outcome (e.g., social—culture), as well as evaluation method, metrics, duration, and frequency.

**Table 12:** Metadata attributes to be extracted during data coding for articles that pass both title and abstract and full text screening stages.

Category	Attribute name	Description
General	Article ID	Unique identifier for each article
	Full text eligibility	Whether article is eligible for inclusion based on full text screening (include, exclude)
	Screener name	Name of individual who screened full text
	Screening date	Screening date of full text
	Full text available	Whether full text is available for article (available, unavailable)
	Screening notes	Screener notes on full text screening stage
Bibliographic	Publication type	Type of publication (peer-reviewed, book chapter, etc.)
	Author(s)	Article author(s)
	Publication year	Year article was published (YYYY)
	Title	Title of article
	Journal name	Name of journal where article was published
	Volume	Volume of journal in which article was published
	Page numbers	Page numbers of article
	DOI	DOI of article
	URL	URL of article
Population	Population eligibility	Inclusion versus exclusion decision during full text screening stage for population (include, exclude)
	Type of coastal ecosystem	Type of coastal ecosystem where NBS is located (salt marsh, seagrass, mangrove, kelp, coral reef, shellfish reef)
	Description of coastal ecosystem	Description from article of ecosystem type
Intervention	Intervention eligibility	Inclusion versus exclusion decision during full text screening stage for intervention (include, exclude)
	Category of NBS intervention	Category of NBS intervention (living shoreline, planting or seeding, artificial substrate addition); see intervention typology, Table 7
	Coastal protection context of NBS intervention	Whether the NBS intervention seeks to achieve a stated coastal protection goal (stated goal; evaluated outcome)
	Policy-relevant term for intervention	Policy-relevant term used to describe NBS intervention (NBS, NBI, NNBF, etc.)
	Description of NBS intervention	Description from article of NBS intervention including quantitative or qualitative information
	Description of NBS' coastal protection goal	Description from article of coastal protection goal that NBS intervention seeks to achieve
	Coastal protection context	Whether coastal protection was identified as an intended goal or intent, evaluated outcome, or implied through framing (intended, assessed, implied)
	Dates of NBS intervention	When the NBS intervention takes place
	Cost of NBS intervention reported	Whether cost information on NBS intervention is reported (yes, no)
Study type	Study type eligibility	Inclusion versus exclusion decision during full text screening stage for study type (include, exclude)
	Study type	Type of study (experimental, modeling, etc.)
	Study objective	Description from article of study objective
	Study design	Description from article of study design
	Study location	Description from article of study location
	Geographic scale	Description from article of NBS intervention geographic scale (global, regional, national, subnational, local)
	Country	Country where NBS intervention occurred
	State	If NBS intervention used in United States, state where NBS intervention occurred
	Water body	Name of water body where study was conducted
	Category of comparator	Category of comparator used in study (before vs. after, presence vs. absence, different types of NBS interventions, etc.)
	Description of comparator	Description from article of study comparator, if applicable
Outcome	Outcome eligibility	Inclusion versus exclusion decision during full text screening stage for outcome (include, exclude)
	Category of outcome	Whether performance outcome is ecological, physical, economic, or social (ecological, physical, economic, social)
	Subcategory of outcome	Subcategory of outcome (social: safety and security, culture, etc.); see outcome typology, Tables 8, 9, 10, 11
	Description of outcome	Description from article of performance outcome
	When outcome evaluation took place	Whether NBS performance outcomes were evaluated before, during, or after construction [before construction, during construction, after construction (≤ 1 yr), after construction (> 1 to ≤ 5 yrs), after construction (> 5 to ≤ 10 yrs), after construction (> 10 yrs), no evaluations conducted]
	Frequency of outcome evaluation	Frequency of outcome performance evaluation, including units (e.g., every 3 weeks for 5 years)
	Methods for outcome evaluation	Monitoring method(s) used to evaluate NBS performance outcome (net sampling, economic survey, etc.)
	Metrics for outcome evaluation	Monitoring metric(s) used to evaluate NBS performance outcome (fish biomass, job creation, etc.)
	Data type for outcome evaluation	Whether data are qualitative, quantitative, or a combination of both to monitor performance outcome
	Directionality outcome	Directionality of performance outcome (positive, negative, mixed, neutral/no effect)

For each attribute, its name and description are provided, along with the category that it falls within (bibliographic, intervention, etc.). If an article has more than outcome, outcome attributes like outcome category and subcategory, outcome description, etc. will be repeated for each outcome

We have developed a code book that explains the metadata attributes (Additional file 6). The code book provides instructions for screeners, designates attribute types and formats, and specifies levels for categorical attributes from which screeners can select from dropdown menus when entering data into the standardized data coding spreadsheet. For attributes where the required information is missing from or not stated in the article, screeners will code the attribute as “unknown.” We do not plan to contact authors to request missing information. When an attribute is not applicable to a particular article, it will be coded as “not applicable.” We will fully test the code book prior to data coding and will report any modifications to the codebook in the final systematic map.

To ensure consistency in data coding, we will hold a training session to train screeners in how to conduct metadata coding; this training session may occur within the full text screening training session (see Screening section above). During the training session, we will collaboratively work through data coding of several articles, including some that are straightforward and others that are more nuanced. We will then assign each screener the same small subset of articles to code. We will compare coding results, discuss inconsistencies, and may alter attributes and instructions if needed. Given the high number of expected articles, we will not conduct double (or side-by-side) data extraction at the full text stage but rather will conduct spot checks on a small percentage of articles. We will compare spot checking results and discuss any inconsistencies with the screening team. The exact percentage of articles for which spot checking will be conducted will depend on the number of total articles, and we will report this information in the systematic map.

## Study mapping and presentation

Metadata extracted from studies that pass title and abstract and full text screening will be converted into a standardized format suitable for analysis. Analyses will be conducted in R to investigate and visualize patterns in the distribution and abundance of evidence surrounding NBS performance. Analyses will be targeted to address our primary and secondary research questions. For example, we will characterize the distribution of evidence on the intended performance of NBS, including the types of coastal protection goals, number of coastal protection goals identified, whether coastal protection goals are most frequently primary goals or co-benefits, and what categories other goals fall under (economic, social, ecological). We will also summarize evidence on the performance of NBS, including the distribution of evidence across ecological (e.g., species and population, biological interactions, nutrient cycling), physical (e.g., water level, waves, sediment and morphology), social (e.g., human health, culture, safety and security), and economic (e.g., income, livelihoods, natural capital) outcomes. We will then determine differences in the evidence base by factors, such as ecosystem type (e.g., salt marsh, mangrove, shellfish reef), NBS type (e.g., system restoration or enhancement, system creation, structure addition), geographic location, and spatial scale. We will identify approaches used to evaluate NBS performance, which metrics were evaluated, and when evaluations were collected relative to the NBS intervention. We will identify topics and subtopics where sufficient evidence exists, termed evidence clusters, suitable for future systematic reviews or meta-analyses. We will also identify evidence gaps suitable for future empirical research. Evidence clusters and gaps will be identified using heat maps based on matrices of the number of studies for cross-tabulated attributes (e.g., interventions versus outcomes).

Following data analyses, we will prepare the final evidence map for peer-reviewed publication in the journal *Environmental Evidence.* The evidence map will include visual summaries of the evidence base using figures including heat maps, bar plots, and geographic distribution maps, as well as tabular summaries. A core component of the map will be a narrative summary highlighting evidence clusters for which systematic reviews or meta-analyses can be conducted, as well as evidence gaps for which additional research may be warranted. The narrative report will also outline the policy and management implications of the map findings. Data on included literature and associated metadata, as well as excluded literature, will be made publicly available either through a public data repository or as Additional files published with the resulting evidence map.

## Supplementary Information

**Additional file 1.** ROSES for systematic map protocols checklist.

**Additional file 2.** Search strategy development and testing.

**Additional file 3.** Benchmarking articles.

**Additional file 4.** Intervention typology.

**Additional file 5.** Outcome typology.

**Additional file 6.** Data extraction codebook.

## Data Availability

Not applicable.
